# Contribution of genetic variation to transgenerational inheritance of DNA methylation

**DOI:** 10.1186/gb-2014-15-5-r73

**Published:** 2014-05-29

**Authors:** Allan F McRae, Joseph E Powell, Anjali K Henders, Lisa Bowdler, Gibran Hemani, Sonia Shah, Jodie N Painter, Nicholas G Martin, Peter M Visscher, Grant W Montgomery

**Affiliations:** 1 The Queensland Brain Institute, University of Queensland, Brisbane, Australia; 2 University of Queensland Diamantina Institute, University of Queensland, Translational Research Institute (TRI), Brisbane, Australia; 3 QIMR Berghofer Medical Research Institute, Brisbane, Australia

## Abstract

**Background:**

Despite the important role DNA methylation plays in transcriptional regulation, the transgenerational inheritance of DNA methylation is not well understood. The genetic heritability of DNA methylation has been estimated using twin pairs, although concern has been expressed whether the underlying assumption of equal common environmental effects are applicable due to intrauterine differences between monozygotic and dizygotic twins. We estimate the heritability of DNA methylation on peripheral blood leukocytes using Illumina HumanMethylation450 array using a family based sample of 614 people from 117 families, allowing comparison both within and across generations.

**Results:**

The correlations from the various available relative pairs indicate that on average the similarity in DNA methylation between relatives is predominantly due to genetic effects with any common environmental or zygotic effects being limited. The average heritability of DNA methylation measured at probes with no known SNPs is estimated as 0.187. The ten most heritable methylation probes were investigated with a genome-wide association study, all showing highly statistically significant *cis* mQTLs. Further investigation of one of these *cis* mQTL, found in the MHC region of chromosome 6, showed the most significantly associated SNP was also associated with over 200 other DNA methylation probes in this region and the gene expression level of 9 genes.

**Conclusions:**

The majority of transgenerational similarity in DNA methylation is attributable to genetic effects, and approximately 20% of individual differences in DNA methylation in the population are caused by DNA sequence variation that is not located within CpG sites.

## Background

DNA methylation is a crucial epigenetic mark associated with regulation of regulating cellular processes including the silencing of gene expression, differentiation and maintaining genomic stability [1]. A growing number of human diseases have been found to be associated with defects in DNA methylation [2,3]. Importantly, DNA methylation (along with other epigenetic changes) provides a biological link between an individual’s environmental exposures and their phenotype.

Despite the important role DNA methylation plays in transcriptional regulation, the transgenerational inheritance of DNA methylation is not well understood. Two forms of inheritance of epigenetic state have been demonstrated: genetic inheritance and epigenetic inheritance. With genetic inheritance, an individual’s underlying DNA sequence affects epigenetic state, with the extreme example being a genetic variant at a CpG locus that can disrupt DNA methylation at this site. Less understood is the role that DNA sequence variation outside of the CpG locus plays in the observed variation in DNA methylation. Epigenetic inheritance is the sequence independent transmission of epigenetic marks across generations and can occur through the incomplete erasure of epigenetic marks during the two major epigenetic reprogramming events that happen at the gamete and zygote stages. Examples of epigenetic inheritance in the mouse include the *agouti viable yellow* (*A*^*vy*^) and *axin-fused* (*Axin*^*Fu*^) alleles [4,5]. Demonstration of epigenetic inheritance in humans remains unsubstantiated, but is supported through circumstantial evidence such as epidemiological studies noting the effect of grandparental food supply on body size and mortality in their grandchildren [6-8]. The relative importance of genetic inheritance, epigenetic inheritance and common environmental influences to locus specific DNA methylation similarity among relatives has not been well estimated on a genome-wide scale.

A number of studies based on limited numbers of twin pairs have shown significantly higher genome-wide average correlations in DNA methylation measures in monozygotic (MZ) twins compared to dizygotic (DZ) twins, indicating a significant genetic component underlying variation in DNA methylation [9]. Studies using the Illumina HumanMethylation27 array found average estimates of heritability of 0.18 and 0.19 in whole blood [10] and 0.12, 0.07 and 0.05 in cord blood mononuclear cells, umbilical vascular endothelial cells and placenta, respectively [11]. Both these studies used fewer than 20 of each MZ and DZ pairs in the estimation of heritability. The differences between average correlations in DNA methylation for MZ and DZ pairs broadly confirmed that observed in previous studies conducted with fewer CpG sites [12,13]. The case for genetic heritability of DNA methylation is also confirmed by genome-wide association studies locating a number methylation quantitative trait loci (mQTL) in both *cis* and *trans* locations [10,14-17].

The interpretation of differences in correlations between MZ and DZ twin pairs is subject to assumptions [18] that are potentially violated when dealing with epigenetic data. In particular, the higher correlation between MZ twin pairs could be partially due to a common epigenentic starting point in MZ twins at the zygotic stage [19]. It is also unclear whether the assumption of equal common environmental influence on the trait in both MZ and DZ twins is applicable to the analysis of DNA methylation due to the different intrauterine environment experienced by the types of twins, in terms of both chorionicity and implantation, and the role of intrauterine environment on shaping the neonatal epigenome [11].

In this study, we investigate the role of genetic heritability in the similarity of DNA methylation between generations using a family based sample of 614 individuals from 117 families consisting of twin pairs, their parents and siblings using DNA methylation measures on peripheral blood lymphocytes typed on Illumina HumanMethylation450 arrays. This allows us to assess the extent of transgenerational inheritance of DNA methylation caused by genetic heritability.

## Results

### The majority of the similarity in DNA methylation levels between relatives is due to genetic effects

Average correlations across probes between relative pairs are given in Table 1. These correlations are the average across all 417,069 probes and thus are estimated with a very small standard error, although the number of pairs for some relationship classes is low. The DZ twin correlation was 0.109 and the MZ twin correlation of 0.200 was roughly twice that value. The DZ correlation was slightly higher than the (non-twin) sibling and parent-offspring correlations suggesting a potential minor common environmental effect for twins. A small correlation of 0.023 was also observed between the (unrelated) parents. As the study design randomised batch effects, this supports a minor common environmental effect on the scale of the nuclear family. The sibling and parent-offspring correlations of 0.090 and 0.089, respectively, were very similar as expected under a genetic inheritance model as parents and offspring share 50% of their genome and siblings share 50% of their genome on average. These results also indicate that the correction for age removed any potential cross generational effects in the data. Splitting the parent-offspring pairs based on parental sex shows a slightly higher correlation between mother-offspring pairs compared to father offspring, indicating some maternal effects on DNA methylation. The average correlation between unrelated pairs is essentially zero. The slight negative correlation is caused by the use of all possible unrelated pairs in calculating the correlation introducing a small bias due to non-independence of pairs. Overall, these data are consistent with the hypothesis that the correlations in DNA methylation between relatives are largely caused by underling genetic similarity, with some limited evidence for common environmental effects in nuclear families and twin pairs. In other words, approximately 20% of individual differences in DNA methylation variation in the population are due to sequence based DNA variants, and they cause the observed resemblance between relatives.

**Table 1:** Average correlation across all probes of normalised methylation measurements between relative pairs

**Relationship**	**Pairs (n)**	**Correlation**	**Expected**^**a**^
MZ twins	67	0.200	*h*^2^
DZ twins	111	0.109	*h*^2^/2
Siblings	262^b^	0.090	*h*^2^/2
Parent-Offspring	362^b^	0.089	*h*^2^/2
Mother-Offspring	190	0.097	*h*^2^/2
Father-Offspring	172	0.085	*h*^2^/2
Parent-Parent	58	0.023	0
Unrelated	187,331^b^	-0.002	0

^a^The expected correlation under an additive genetic model with a heritability of *h*^2^.

^b^This is the number of quasi-independent pairs as some individuals are represented in multiple pairings.

**Figure 1:**
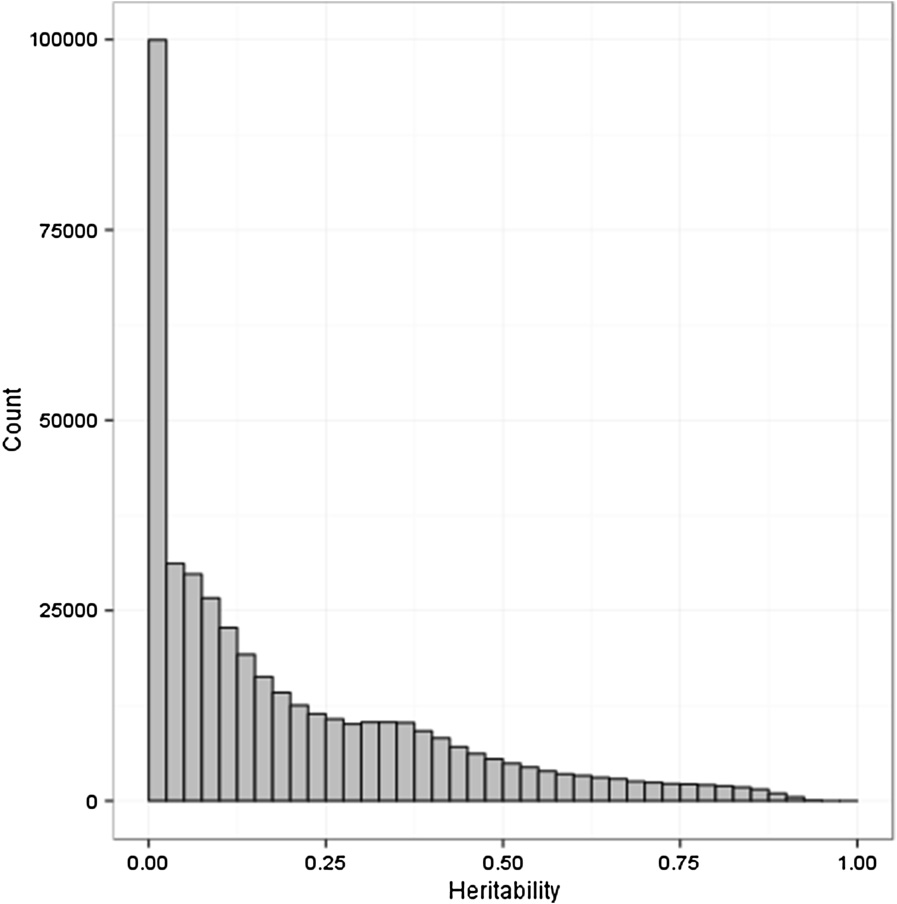
**Distribution of heritability estimates for DNA methylation levels.** The average genetic heritability estimate is 0.199. A zero estimate for genetic heritability was observed in 17.1% of cases indicating that genetic heritability results in transgenerational inheritance of DNA methylation for at least 65.8% of probes.

Estimating the genetic heritability at each measured DNA methylation probe gave an average genetic heritability of 0.199 (Figure 1). Estimated heritabilities at each probe are given in Additional file 1: Table S1. As the estimation was performed using maximum likelihood, there is a lower bound on the heritability estimates of zero. In our data, we observed 17.1% of the probes to give an estimated genetic heritability of zero. Under the null hypothesis of no heritable genetic component to DNA methylation, we would expect 50% of the probes to give a zero estimate and our data provide strong evidence for a significant genetically heritable component to variation in DNA methylation. We can also use the proportion of zero results to provide a lower bound to the proportion of DNA methylation probes with a genetic component to the variation of 65.8% (=100% - 2 × 17.1%). At a 5% Benjamini-Hochberg false discovery rate, 202,162 (48.5%) probes show significant genetic heritability. In addition, we performed sensitivity analysis that confirmed the heritability estimates were not biased upwards by potential batch effects (Additional file 2: Figure S2), consistent with the design of our experiment.

It is likely that a proportion of probes on the array show no variation in DNA methylation in peripheral blood lymphocytes. While there was no clear cutoff observed in the between those probes with only experimental noise in their DNA methylation level estimates and those with low levels of genuine DNA methylation variation, we can examine the effect of non-variable positions by comparing probes with high levels of observed variation to those with low levels (Additional file 3: Figure S3). The probes with the lowest observed variation show a reduction in their average estimated heritability. However, excluding the 25% of probes with the lowest observed variation only increases the average estimated heritability to 0.212 from 0.199, demonstrating any bias introduced by including probes in regions with invariant DNA methylation is limited.

We estimated the effect of environment on the similarity of DNA methylation by fitting a common environment effect for the nuclear families in addition to the additive genetic effect. Given the nuclear family design used in this study, the effects of common environment and epigenetic inheritance are highly confounded and so difficult to estimate separately. Thus the estimated common environmental effect will be inflated by potential epigenetic inheritance. The estimated common environmental variance was small, with an average of 2.3%. Overall, 56% of sites had an estimated zero common environmental variance. While this is greater than the 50% expected under the null hypothesis, attributable to the bias created when trying to separate out the correlated additive genetic and common environment effects in this sample size, it does provide a strong indication that the common environment of a nuclear family explains very little variation in DNA methylation levels. Similarly, none of the probes show a significant common environmental variance at a Benjamini-Hochberg false discovery rate of 5%.

### Effect of cellular composition on heritability estimates

It has been shown that at some loci the DNA methylation level when measured from peripheral blood leukocytes is reflective of the underlying cell composition. To exclude this as a driving force behind the heritability estimates, we estimated the proportion of monocytes, B cells, natural killer, CD4+ and CD8+ T-cells and granuloctyes from the DNA methylation data [20]. We then included the estimated proportion of each of these cell types as covariates while estimating the heritability of each DNA methylation probe. Correcting for the cellular make-up reduced the average heritability estimate of DNA methylation by 0.199 to 0.176. As shown in Additional file 4: Figure S4, the cellular composition of the samples had little effect on the heritability estimates for the majority of probes investigated. In particular, the heritability estimates for probes showing high heritability were relatively stable. Probes with lower heritability showed more bias due to cellular composition, although this was limited to a subset of approximately 7% of the probes.

### Excluding SNPs effects on genetic heritability estimates of DNA methylation

Using the 1000 Genomes Phase I Version 3 data from European individuals [21], the number of SNPs and their location in each probe was investigated. Additional file 5: Figure S5 shows that the average methylation heritability substantially increases with the number of SNPs within a probe region. This increase in DNA methylation genetic heritability can be attributed to genuine DNA methylation differences caused by SNPs at the CpG site or through *cis* genetic effects, or alternatively to a SNP causing differences in binding of alternative alleles to the array. This effect is further investigated in Additional file 6: Figure S6 where the average genetic heritability is correlated with the position on the SNP in the probe for all probes containing a single known SNP. It is evident that the primary increase in genetic heritability is when the SNP is within the CpG site, although this effect does extend across the whole probe with the average genetic heritability being greater than that observed in probes that do not overlap known SNPs. To avoid potential biases in estimates of genetic heritability due to effects of SNPs on array binding, we removed all probes with known SNPs from the dataset. The average genetic heritability of the remaining 303,078 probes was 0.187, slightly less than the estimate of 0.199 obtained including all probes. Of this subset, 141,596 (46.7%) were significantly genetically heritable at Benjamini-Hochberg false discovery rate of 5%.

### The effect of genomic context on genetic heritability of DNA methylation

The role of genomic context on the genetic heritability of DNA methylation was investigated by separating probes into categories based on the density of DNA methylation. The ‘HIL’ classification as defined in [22] was used, which categorises probes into those found in high density CpG islands (HC), intermediate density CpG island (IC) and non-island (LC), with intermediate density group is further separated out into those intermediate-density probes that border high-density islands (ICshore). Probes in high density regions had a reduced genetic heritability compared to those in intermediate and low density regions which showed similar genetic heritability levels (Table 2). This reduction of average heritability in high density regions was not explained by a higher portion of those probes measuring regions with invariant DNA methylation levels, with the observed variation at the high-density probes being on average higher than the other classes. A higher number of CpG sites within a probe correlated with a lower average genetic heritability (Additional file 7: Figure S7). This effect also accounted for much of the difference in average heritability between the two different probe types on the Illumina HumanMethylation450 array (Type I average h^2^ = 0.154 and Type II h^2^ = 0.198) as the probe types interrogate sites with different average numbers of CpGs.

**Table 2:** **Differences in average heritability for different ‘HIL’ categories of the measured CpGs **[22]** separated by the two probe types on the Illumina HumanMethylation450 array**

**Probe classification**	**Average heritability estimate**	
	**Type I probes**	**Type II probes**
HC	0.127 (61,718)	0.158 (71,817)
ICshore	0.220 (7,822)	0.241 (22,192)
IC	0.223 (28,467)	0.223 (68,438)
LC	0.235 (7,722)	0.223 (148,893)

The probes are categorised into those found in high-density CpG islands (HC), intermediate-density CpG island (IC) and non-island (LC), with intermediate-density group is further separated out into those intermediate-density probes that border high-density islands (ICshore). The number of probes in each category is provided in brackets.

**Figure 2:**
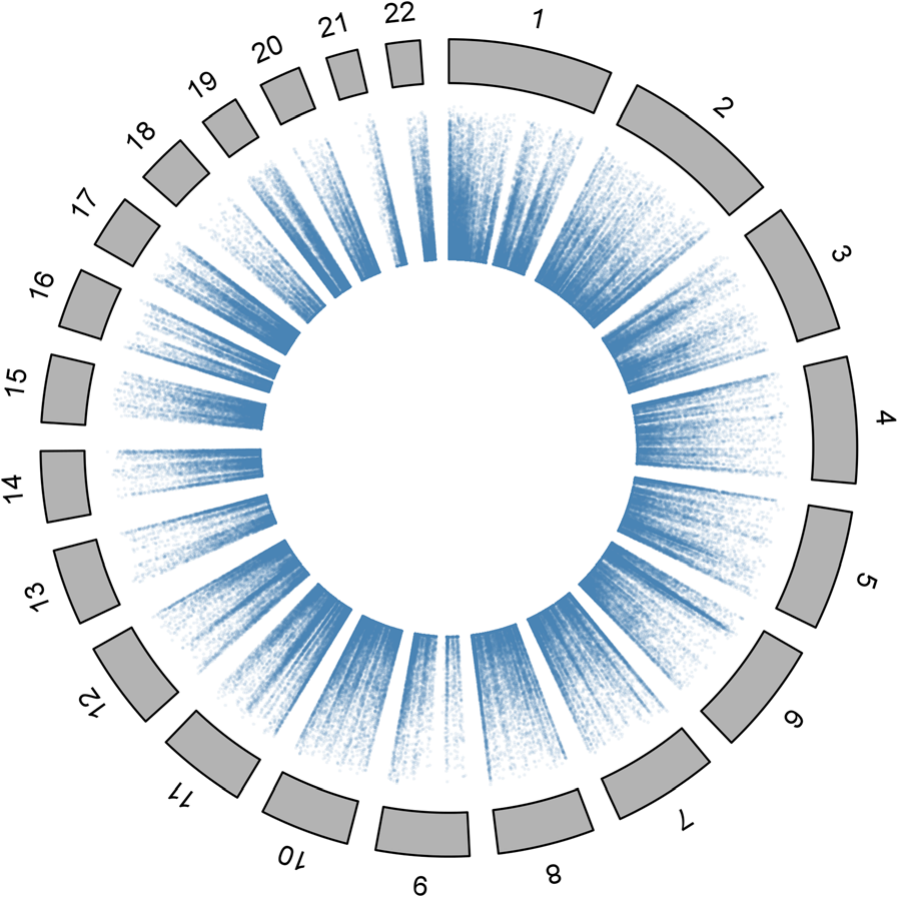
**Distribution of genetic heritability estimates across the genome.** The MHC region, which had the highest estimates of genetic heritability is clearly visible on chromosome 6. Telomeric regions show an increased density of probes with high genetic heritability, although this is primarily due to higher numbers of probes in these regions.

Figure 2 shows the distribution of genetic heritability estimates across the genome, demonstrating probes with high estimated heritabilities are located throughout the genome. The apparent increase in genetic heritability in the telomeric regions is primarily an artefact caused by the higher density of DNA methylation probes on the array in these regions compared to the rest of the genome resulting in a greater number of probes with high estimated DNA methylation heritability. However, there is also a small increase in the average heritability for those probes within 1Mpb of the telomere (average h^2^ = 0.217 *vs.* h^2^ = 0.186).

### Identification of complex genetic associations with DNA methylation and gene-expression

**Figure 3:**
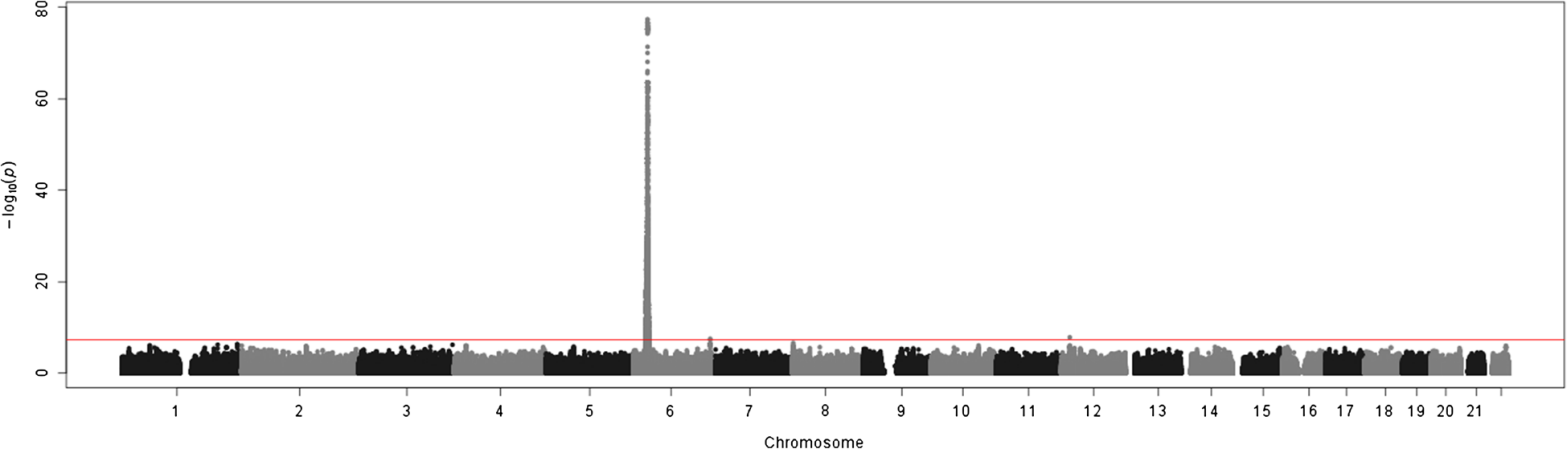
**Manhattan plot of the genome-wide association *P *values for methylation probe cg15671450.** The genome-wide significance level of 5 × 10^-8^ is indicated by the horizontal line. A highly significant effect is observed *cis* to the methylation probe on chromosome 6.

**Figure 4:**
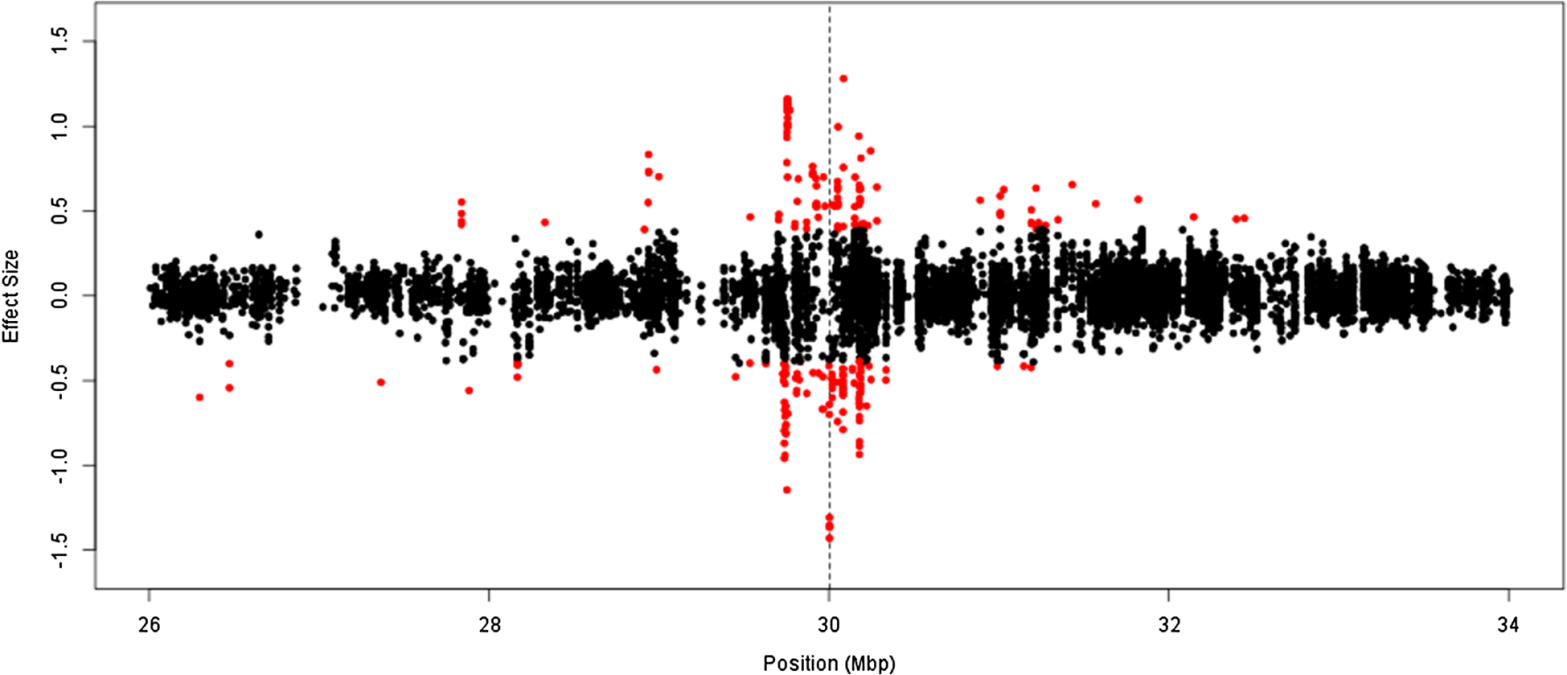
**Association between rs111482415 and DNA methylation probes in the surrounding 8 Mbp window.** The effect size measures the change in the log-odds of the probe being methylated with changing genotype, with positive values indicating an increased average methylation level. Probes with a significant association to rs111482415 at a genome-wide Bonferroni corrected 0.05 level are coloured red. The position of rs111482415 is indicated with a dashed line. See also Additional file 9: Figure S8 and Additional file 8: Table S2.

A genome-wide association study was performed for the 10 most heritable probes without a known SNP in their probe, which covered a range of genomic contexts in terms of probe type, ‘HIL’ classification and CpG content (Table 3). All probes have a highly significant *cis* mQTL. Figure 3 gives an example Manhattan plot for the most highly heritable probe, cg15671450, located in the MHC region of chromosome 6. Testing the most significant associated SNP, rs111482415 (located in the HLA region), for association with DNA methylation probes in the surrounding 8 Mbp region found significant association with 209 other probes at a genome-wide significant Bonferroni level of 1.2 × 10^-7^ (0.05/417,069), with associations observed in both the positive and negative direction (Figure 4). Testing for association between the SNP rs111482415 and gene-expression levels measured in peripheral blood lymphocytes on a cohort of which the samples used in this study are a subset [23] found nine genome-wide significant associations, all located within the same region (Additional file 8: Table S2).

**Table 3:** Details of the 10 most heritable probes that do not contain any annotated SNPs

**Probe ID**	**Chr**	**CpG position**	**h**^**2**^	**Genomic context**	**Type**	**HIL**	**#CpG**	**GWAS SNP**	**SNP position**	***P *** **value**
cg15671450	6	29895116	0.934	Upstream (HCG4B)	II	HC	1	rs111482415	29923140	4.8 × 10^-78^
cg01903420	13	27295928	0.933	Intergenic	II	IC	2	rs1374010	27295317	3.0 × 10^-105^
cg03168497	17	48586147	0.932	Intronic (MYCBPAP)	II	HC	4	rs73351675	48585554	8.1 × 10^-84^
cg11064039	7	766100	0.932	Intronic (PRKAR1B)	I	HC	3	rs11763218	852281	8.8 × 10^-58^
cg24372256	21	43528868	0.931	Intronic (UMODL1)	II	IC	1	rs34212454	43529216	2.9 × 10^-101^
cg26764761	16	87682142	0.927	Intronic (JPH2)	I	IC	7	rs748554	87682775	1.4 × 10^-107^
cg16761754	14	105127242	0.927	Intergeneic	I	IC	3	rs4075355	105125512	1.8 × 10^-77^
cg21358336	17	6558440	0.927	Upstream (MIR4520B)/Downstream (MIR4520A)	II	ICshore	1	rs2040847	6558011	1.3 × 10^-91^
cg04118610	4	62707027	0.926	Intronic (LPHN3)	II	LC	2	rs10021525	62707476	2.1 × 10^-105^
cg08164151	12	131118432	0.925	Intergeneic	II	IC	3	rs10848167	131123623	2.9 × 10^-101^

Genomic context of probes was annotated using ANNOVAR [24], with a probe being upstream or downstream defined as being within 2 KB of the transcription start site or transcription end site, respectively.

GWAS SNP = Most significant SNP from GWAS; HIL = ‘HIL’ classification of CpG [22]; Type = Illumina HumanMethylation450 assay probe type.

Additional file 9: Figures S8a-i provide equivalent plots for the 8 Mbp region surrounding the most significant GWAS SNP for the remaining nine of the 10 most heritable probes. Association signals for these SNPs extended only a few hundred kbp into the surrounding region with one exception. SNP rs10021525 was associated with methylation measured by a single probe. Of the other SNPs, rs10848167 was significantly associated with 109 probes at a genome-wide Bonferroni corrected level. The majority of SNPs showed associations with effects on methylation in both increasing and decreasing directions. None of these nine SNPs were significantly associated with gene-expression after correction for genome-wide multiple testing.

## Discussion and conclusions

We have demonstrated significant genetic control for the transgenerational inheritance of DNA methylation. Of particular importance, genetic heritability is shown to be the major cause of similarity in DNA methylation levels among relatives when considering the average across the genome. This study conclusively demonstrates that previous small scale studies on twin pairs [10,11] are not greatly biased in their genetic heritability estimates through potential zygotic effects due to MZ twins starting from a common epigenetic state [19]. However, estimates for twin pairs were observed to be slightly more similar to each other than to their siblings, indicating that small environmental or zygotic effect exists, although the contribution to similarity among relatives is at least an order of magnitude less than the estimated genetic heritability. Similarly, while direct testing failed to detect any significant common environmental influence on DNA methylation on the scale of the nuclear family, there was a small non-zero average parent-parent correlation observed indicating that such effects may be found in larger studies. This small correlation between parents may suggest some aspect of assortative mating, although these families show no evidence for assortative mating based on DNA sequence variant information [23]. It is also plausible that the parent-parent correlation could be explained by unaccounted for experimental artefacts, as while the plating position and batch for each individual was completely randomised on the DNA methylation arrays, family groups tended to have blood collected at the same time.

Investigating epigenetic inheritance in an outbred population is difficult. When fitting a mixed linear model to nuclear families such as those used in this study, it is particularly difficult to separate out common environmental effects from potential epigenetic inheritance. As these effects are confounded, the observation of no significant common environmental effects observed in our data suggests that epigenetic inheritance is not widespread at these loci. However, this is not a directly test for epigenetic inheritance and a number of different models (involving no correlation between the spouses and potentially inheritance occurring through only the maternal or paternal lines) could be formed to test this. Using this approach to detect epigenetic inheritance would require a very large sample size in order to separate the various competing models for the action of both epigenetic inheritance and common environmental effects.

A potential bias to estimating genetic heritability of DNA methylation in arrays is the role of SNPs in the probe locations. While some SNPs cause genuine DNA methylation differences - such as those found in the CpG site - they may also affect the binding of the probe to the array. The effect of SNPs within probe sequences was minimised by using data from the European cohort of the 1000 Genomes Project [21] and excluding all probes with known variants (approximately 27%). Given the evidence for *cis* genetic effects on DNA methlylation, this is probably too stringent a filter in that it removes genuine heritable genetic variation in DNA methylation. It is likely that not all genetic sequence variation in the probe regions has yet been detected in the 1000 Genomes Project. However, given the effect of SNPs within the probes on average heritability was shown to be limited and any non-detected variant will be at low frequency in the population, such variants will have only a small effect on the heritability estimates presented.

A GWAS of the 10 most genetically heritable DNA methylation probes in our data found large *cis* mQTL. Notably, the most heritable DNA methylation probe is located in the MHC region, which is known to be associated with a wide range of diseases and other complex traits [25] providing potential insight to the biological mechanisms underlying these associations. The top SNP at this mQTL was associated with more than 200 DNA methylation probes in the surrounding 8 Mbp region and also with expression at 10 genes. The length of the associated region was unique among the cis mQTL the top 10 most heritable probes, but a more extensive GWAS analysis for all probes across the genome is required to assess the length distribution of genomic regions influenced by mQTLs and whether the MHC region is unique. Also of interest was the bidirectional effect of associations with both increased and decreased DNA methylation and expression levels. Such effects were replicated in many of the other top 10 most heritable DNA methylation probes indicating the complex pattern was not solely due to the known complex linkage disequilibrium structure in the MHC region. It has been demonstrated that DNA methylation can affect gene-expression in either a passive or active manner [26]. Our results show associations both increasing and decreasing DNA methylation and gene-expression and it is difficult to infer a simple biological mechanism behind these associations from this dataset.

In summary, we have provided convincing evidence that the majority of transgenerational similarity in DNA methylation is attributable to genetic effects, and that approximately 20% of individual differences in DNA methylation in the population are caused by DNA sequence variation that is not located within CpG sites.

## Materials and methods

### Study participants

DNA methylation was measured on 614 individuals from 117 families of European descent recruited as part of a study on adolescent twins and selected from individuals in the Brisbane Systems Genetics Study [23]. Families consist of adolescent MZ and DZ twins, their siblings and their parents. This study was approved by the Human Research Ethics Committee of the Queensland Institute for Medical Research. All participants gave informed written consent. DNA was extracted from peripheral blood lymphocytes by the salt precipitation method [27] from samples that were time matched to sample collection of PAXgene tubes for gene expression studies in the Brisbane Systems Genetics Study [23].

### DNA methylation

Bisulfite conversions were performed in 96 well plates using the EZ-96 DNA Methylation Kit (Zymo Research, Irvine, CA, USA). Prior to conversion, DNA concentrations were determined by NanoDrop quantification (NanoDrop Techologies, Inc., Wilmington, DE, USA) and standardised to include 500 ng. Three technical replicates were included in each conversion to assess repeatability. A commercial female human genomic DNA sample (Promega Corporation, Madison, WI, USA) was used on all plates, one sample from each run was duplicated on the plate and one sample duplicated from a different plate. DNA recovery after conversion was quantified using Nanodrop (Thermo Scientific, Wilmington, DE, USA).

Bisulfite converted DNA samples were hybridised to the 12 sample, Illumina HumanMethylation450 BeadChips using the Infinium HD Methylation protocol and Tecan robotics (Illumina, San Diego, CA, USA). The HM 450 BeadChip-assessed methylation status was interrogated at 485,577 CpG sites across the genome. It provides coverage of 99% of RefSeq genes. Methylation scores for each CpG site are obtained as a ratio of the intensities of fluorescent signals and are represented as β-values. Samples were randomly placed with respect to the chip they were measured on and to the position on that chip in order to avoid any confounding with family. DNA methylation data are available at the Gene Expression Omnibus under accession code GSE56105.

Box-plots of the red and green intensity levels and their ratio were used to ensure that no chip position was under- or over-exposed, with any outlying samples repeated. Similarly, the proportion of probes with detection *P* value less than 0.01 was examined to confirm strong binding of the sample to the array. Probes on the sex chomosomes or having been annotated as binding to multiple chromosomes [22] were removed from the analysis, as were those with zero CpG sites. The probability of a probe within a sample either being called as missing or with a detection *P* value less than 0.001 were estimated from the average rate across all probes and samples. A threshold for probes showing significant deviation from random missingness (or excess poor binding) was determined by testing against a binomial distribution for the number of samples at the 0.05 significance level with a Bonferroni correction for the number of probes. Any probe with more than 11 individuals with missing data or more than five individuals with detection *P* values > 0.001 were removed. After cleaning, 417,069 probes remained. A flow chart depicting the data cleaning and the number of arrays and probes removed at each stage is given in Additional file 10: Figure S1.

### Normalisation

No global normalisation was performed on the methylation arrays as, for example, quantile normalisation may remove genetic and environmental effects that act globally on methylation. Individual probes were normalised across all samples using a generalised linear model with a logistic link function. Corrections were made for the effects of chip (which encompasses batch processing effects), position on the chip, sex, age, age^2^, sex × age and sex × age^2^. All heritability analyses were performed using the residuals from this model. No correction for differences between the two probe types was performed as the heritability analysis is partitioning the variation within a particular probe, so the observed shrinking of the Type II probes away from measures of 0 and 1 does not have an effect on the results.

To avoid undue influence of outlying data points (which could either be genuine unique methylation differences or measurement error) on both the estimates of heritability and following GWAS analysis, any measurement greater than five interquartile ranges from its nearest quartile was set to missing. The choice of threshold was determined by comparing heritability estimates with and without outliers included and noting the point at which outliers affected the results (data not shown).

### Heritability estimation

For each probe, the intraclass correlation for the various relative pairs was calculated using ANOVA as:

$$\mathrm{ICC}=\frac{MS_{B}-MS_{W}}{MS_{B}+MS_{W}}$$

Where *MS*_*B*_ is the Mean Square Between pairs and *MS*_*W*_ is the Mean Square Within.

As the relationship pairs indicated that on average common environment effects are small, the heritability for each probe was estimated by partitioning its variance into additive genetic (*V*_*a*_) and environmental (*V*_*e*_) components. Additionally, a model which included a nuclear family common environmental effect (*V*_*f*_) was tested. All models were fitted using QTDT v2.6.1 [28].

### Genome-wide association analysis

Genome-wide association analyses were performed on the 10 most heritable probes without known SNPs within the probe. All individuals were genotyped on Illumina 610-Quad Beadchip arrays. Full details of genotyping procedures are given elsewhere [29]. Standard QC filters were applied, leaving 528,509 SNPs. The remaining genotyped SNPs were phased using HAPI-UR [30] and imputed using 1000 Genomes Phase I Version 3 [21] with Impute V2 [31,32]. Raw imputed SNPs were filtered to remove any SNPs with low imputation quality as defined by an R^2^ < 0.8. Subsequent quality control removed SNPs with MAF <0.05, those with HWE p < 1 × 10^-6^, and a missing rate >10%. After filtering, 6,005,138 SNPs remained for further analysis. Association analysis on the imputed genotype probabilities was performed using Merlin [33].

### Gene expression

Gene expression was measured on peripheral blood lymphocytes using the Illumina HT-12 v4.0 array and the data were normalised as described in detail elsewhere [23]. The individuals used in this study represent a subset of the cohort with gene-expression measurements. After cleaning, 17,926 probes remained for association testing. Gene expression data are available at the Gene Expression Omnibus under accession code GSE53195.

## Competing interests

The authors declare that there are no conflicts of interests.

## Authors’ contributions

Conceived and designed the experiments: AFM, PMV and GWM. Performed the experiments: AKH, LB and JNP. Analysed the data: AFM, JEP, GH and SS. Contributed reagents/materials/analysis tools: NGM. Wrote the paper: AFM, PMV and GWM. All authors read and approved the final manuscript.

## Supplementary Material

Additional file 1: Table S1 Heritability estimates for 417,069 DNA methylation probe measures. The location of the target CpG site is given with Build 37 coordinates, along with the number of SNPs detected in the probe sequence from the European individuals in the 1000 Genomes Phase I Version 3 data. Heritability estimates with and without correction for estimated blood cell composition are provided. Click here for file

Additional file 2: Figure S2 Confirming heritability estimates are not inflated by potential batch effects. Heritabilities were re-estimated for 50,000 probes with the array each individual was measured on included as an additional covariate. As expected from both our study design that randomly placed individuals across arrays and the prior normalisation performed, the heritability estimates marginally increase when including array as a covariate. This does not mean that our reported estimates are biased downwards, but is due to additional random noise being introduced through correcting for array twice in different models. Given our study design, such double correction will bias the estimated environmental variance downwards and therefore the heritability upwards. Click here for file

Additional file 3: Figure S3 Effect of observed variation at probes on the estimated heritability. Probes were ranked and grouped into bins of 1,000 based on the variance of DNA methylation measures after normalization. The probes with the lowest observed variation show a reduction in the average heritability, consistent with these bins containing probes in regions containing no underlying variation in DNA methylation. Click here for file

Additional file 4: Figure S4 Comparison of estimates of heritability with and without correction for estimated cellular composition in the peripheral blood lymphoctye samples analysed. Approximately 7% of probes showed an upward bias in the estimated heritability when not accounting for cellular composition, although probes showing high heritability were relatively robust. Click here for file

Additional file 5: Figure S5 Relationship between the number of known SNPs in a probe and the estimated heritability of DNA methylation. The average estimated heritability increases with the number of SNPs in the European subset of the 1000 Genomes project. Click here for file

Additional file 6: Figure S6 The effect of probe SNP position relative to the target CpG site location on the average estimated DNA methylation heritability for probes with a single annotated SNP. The dotted line indicates the average heritability for probes containing no known SNP. As expected, a substantial effect is observed when a SNP disrupts the target CpG site (position 0). However, the effect of SNPs on the average heritability extends across the entire probe, indicating that SNPs also affect binding to the DNA methylation arrays. Click here for file

Additional file 7: Figure S7 Heritability of DNA methylation and its relationship to the number of CpGs covered by the array probe. The average heritability decreases with an increase in the number of CpGs covered by the probe. This effect accounts for the majority of the difference in average heritability between the two probe types on the Illumina HumanMethylation450 array - Type I (blue) and Type II (red). Click here for file

Additional file 8: Table S2 Genome-wide significant associations between gene-expression levels and rs111482415. Click here for file

Additional file 9: Figure S8 Effect size for the association between most significant SNPs from the GWAS of the 10 most heritable methylation probes and the surrounding DNA methylation probes in the surrounding 8Mbp window. (a) rs1374010 - chromosome 13, (b) rs73351675 - chromosome 17, (c) rs11763218 - chromosome 7, (d) rs34212454 - chromosome 21, (e) rs748554 - chromosome 16, (f) rs4075355 - chromosome 14, (g) rs2040847 - chromosome 17, (h) rs10021525 - chromosome 4, (i) rs10848167 - chromosome 12. Click here for file

Additional file 10: Figure S1 Flow chart detailing the number of samples and probes removed at each step of the DNA methylation array data cleaning. Click here for file
