# Supplementary figures and images for: Contribution of genetic variation to transgenerational inheritance of DNA methylation
Source: Genome Biol. 2014 May 29;15(5):R73. doi: 10.1186/gb-2014-15-5-r73 (PMC4072933; doi:10.1186/gb-2014-15-5-r73)

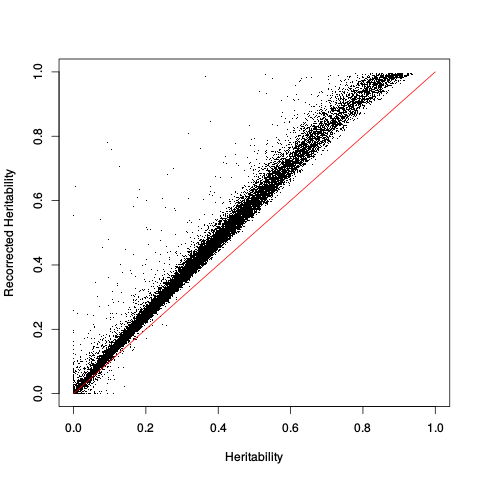

Supplement: Additional file 2: Figure S2 — Confirming heritability estimates are not inflated by potential batch effects. Heritabilities were re-estimated for 50,000 probes with the array each individual was measured on included as an additional covariate. As expected from both our study design that randomly placed individuals across arrays and the prior normalisation performed, the heritability estimates marginally increase when including array as a covariate. This does not mean that our reported estimates are biased downwards, but is due to additional random noise being introduced through correcting for array twice in different models. Given our study design, such double correction will bias the estimated environmental variance downwards and therefore the heritability upwards. [file gb-2014-15-5-r73-S2.png]

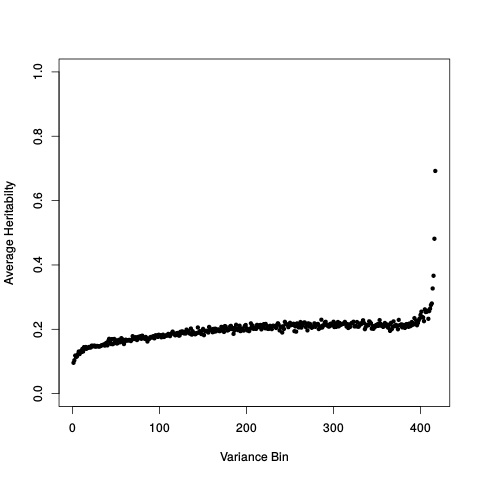

Supplement: Additional file 3: Figure S3 — Effect of observed variation at probes on the estimated heritability. Probes were ranked and grouped into bins of 1,000 based on the variance of DNA methylation measures after normalization. The probes with the lowest observed variation show a reduction in the average heritability, consistent with these bins containing probes in regions containing no underlying variation in DNA methylation. [file gb-2014-15-5-r73-S3.png]

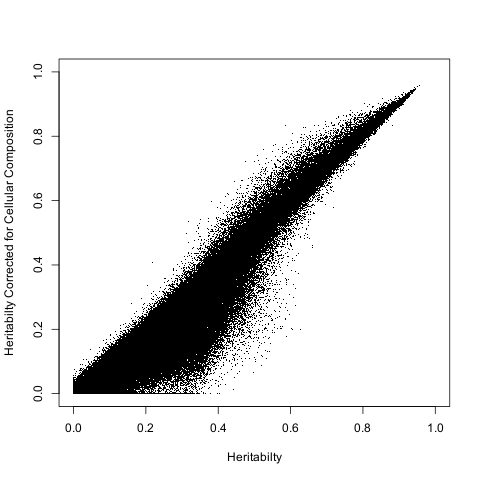

Supplement: Additional file 4: Figure S4 — Comparison of estimates of heritability with and without correction for estimated cellular composition in the peripheral blood lymphoctye samples analysed. Approximately 7% of probes showed an upward bias in the estimated heritability when not accounting for cellular composition, although probes showing high heritability were relatively robust. [file gb-2014-15-5-r73-S4.png]

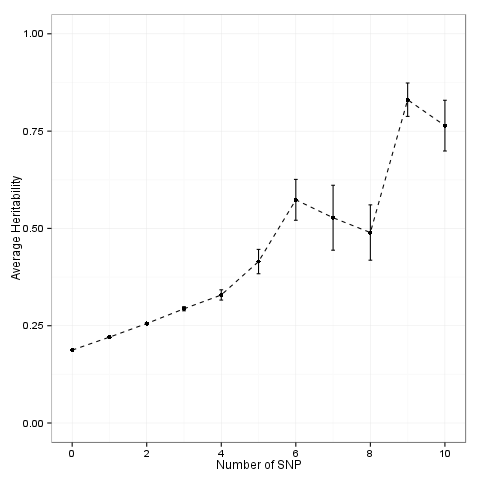

Supplement: Additional file 5: Figure S5 — Relationship between the number of known SNPs in a probe and the estimated heritability of DNA methylation. The average estimated heritability increases with the number of SNPs in the European subset of the 1000 Genomes project. [file gb-2014-15-5-r73-S5.png]

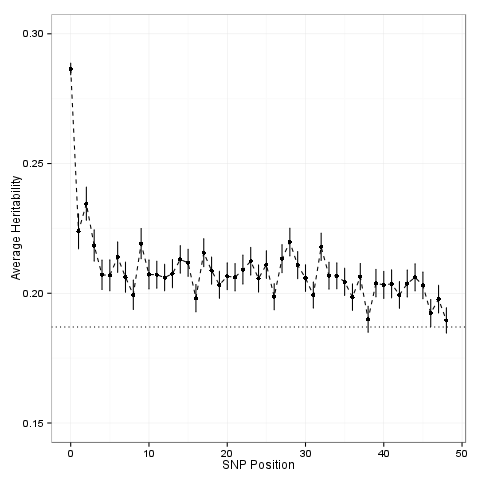

Supplement: Additional file 6: Figure S6 — The effect of probe SNP position relative to the target CpG site location on the average estimated DNA methylation heritability for probes with a single annotated SNP. The dotted line indicates the average heritability for probes containing no known SNP. As expected, a substantial effect is observed when a SNP disrupts the target CpG site (position 0). However, the effect of SNPs on the average heritability extends across the entire probe, indicating that SNPs also affect binding to the DNA methylation arrays. [file gb-2014-15-5-r73-S6.png]

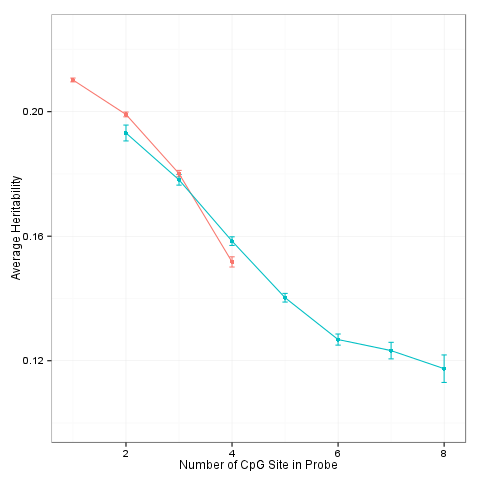

Supplement: Additional file 7: Figure S7 — Heritability of DNA methylation and its relationship to the number of CpGs covered by the array probe. The average heritability decreases with an increase in the number of CpGs covered by the probe. This effect accounts for the majority of the difference in average heritability between the two probe types on the Illumina HumanMethylation450 array - Type I (blue) and Type II (red). [file gb-2014-15-5-r73-S7.png]

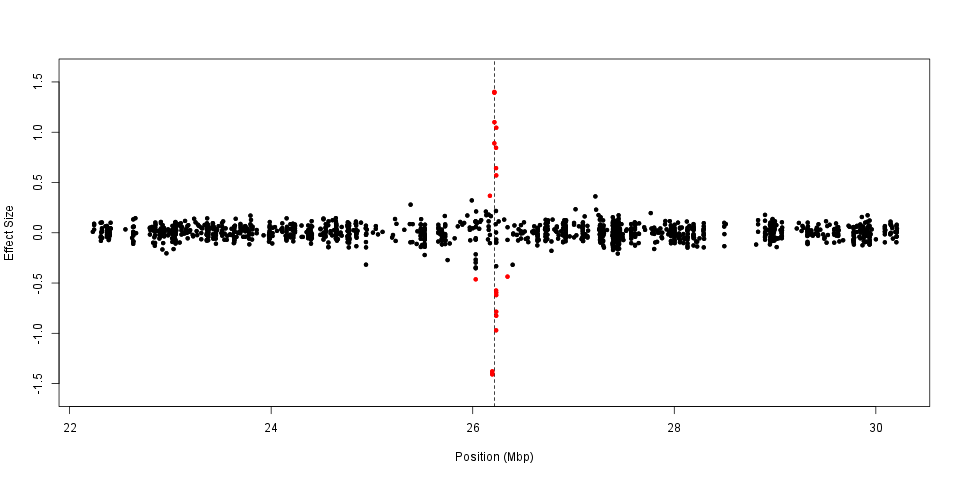

Supplement: Additional file 9: Figure S8 — Effect size for the association between most significant SNPs from the GWAS of the 10 most heritable methylation probes and the surrounding DNA methylation probes in the surrounding 8Mbp window. (a) rs1374010 - chromosome 13, (b) rs73351675 - chromosome 17, (c) rs11763218 - chromosome 7, (d) rs34212454 - chromosome 21, (e) rs748554 - chromosome 16, (f) rs4075355 - chromosome 14, (g) rs2040847 - chromosome 17, (h) rs10021525 - chromosome 4, (i) rs10848167 - chromosome 12. [file gb-2014-15-5-r73-S9.zip › 3001_6051837451123248_MOESM10_ESM.png]

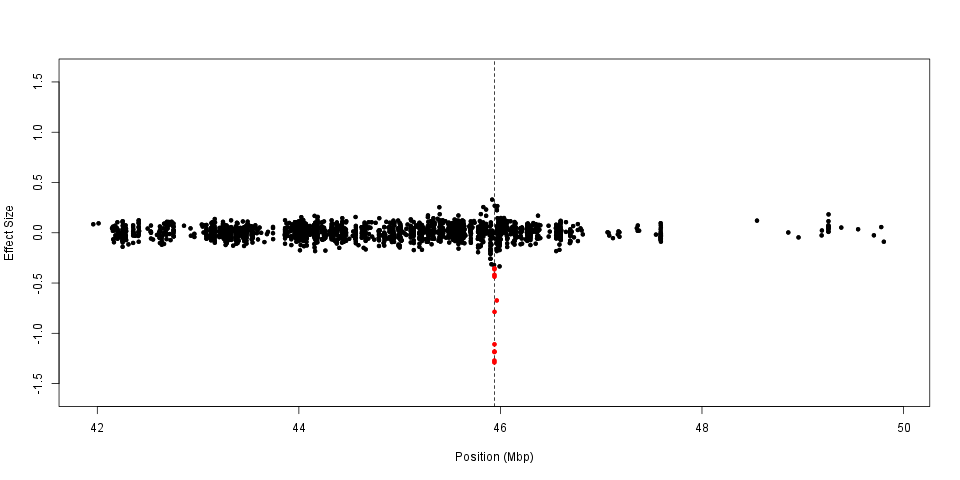

Supplement: Additional file 9: Figure S8 — Effect size for the association between most significant SNPs from the GWAS of the 10 most heritable methylation probes and the surrounding DNA methylation probes in the surrounding 8Mbp window. (a) rs1374010 - chromosome 13, (b) rs73351675 - chromosome 17, (c) rs11763218 - chromosome 7, (d) rs34212454 - chromosome 21, (e) rs748554 - chromosome 16, (f) rs4075355 - chromosome 14, (g) rs2040847 - chromosome 17, (h) rs10021525 - chromosome 4, (i) rs10848167 - chromosome 12. [file gb-2014-15-5-r73-S9.zip › 3001_6051837451123248_MOESM11_ESM.png]

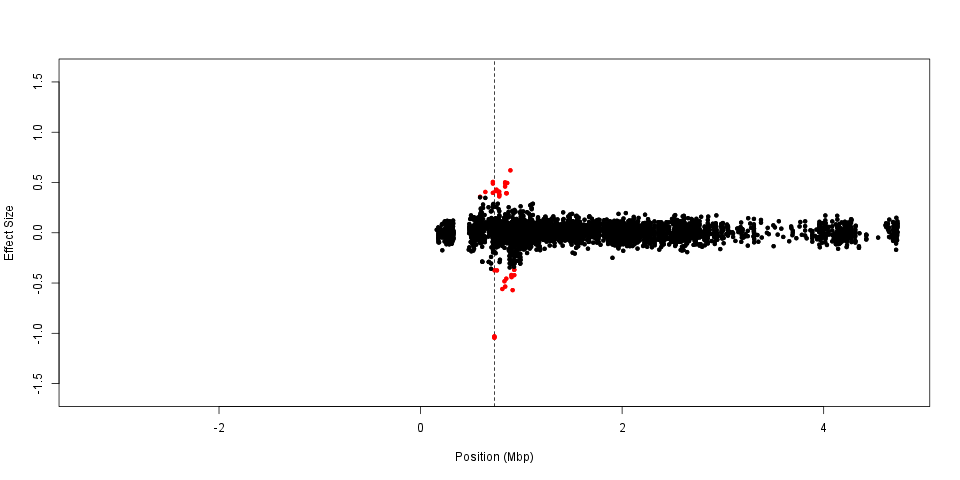

Supplement: Additional file 9: Figure S8 — Effect size for the association between most significant SNPs from the GWAS of the 10 most heritable methylation probes and the surrounding DNA methylation probes in the surrounding 8Mbp window. (a) rs1374010 - chromosome 13, (b) rs73351675 - chromosome 17, (c) rs11763218 - chromosome 7, (d) rs34212454 - chromosome 21, (e) rs748554 - chromosome 16, (f) rs4075355 - chromosome 14, (g) rs2040847 - chromosome 17, (h) rs10021525 - chromosome 4, (i) rs10848167 - chromosome 12. [file gb-2014-15-5-r73-S9.zip › 3001_6051837451123248_MOESM12_ESM.png]

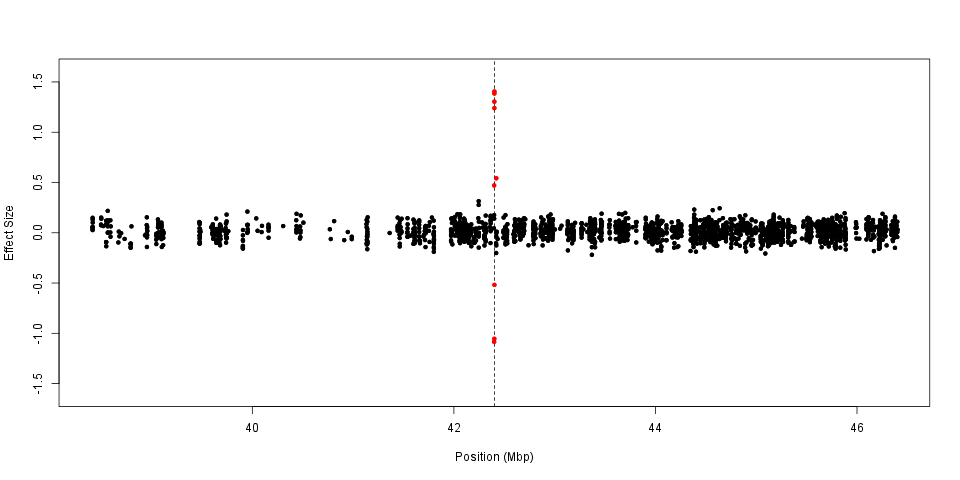

Supplement: Additional file 9: Figure S8 — Effect size for the association between most significant SNPs from the GWAS of the 10 most heritable methylation probes and the surrounding DNA methylation probes in the surrounding 8Mbp window. (a) rs1374010 - chromosome 13, (b) rs73351675 - chromosome 17, (c) rs11763218 - chromosome 7, (d) rs34212454 - chromosome 21, (e) rs748554 - chromosome 16, (f) rs4075355 - chromosome 14, (g) rs2040847 - chromosome 17, (h) rs10021525 - chromosome 4, (i) rs10848167 - chromosome 12. [file gb-2014-15-5-r73-S9.zip › 3001_6051837451123248_MOESM13_ESM.png]

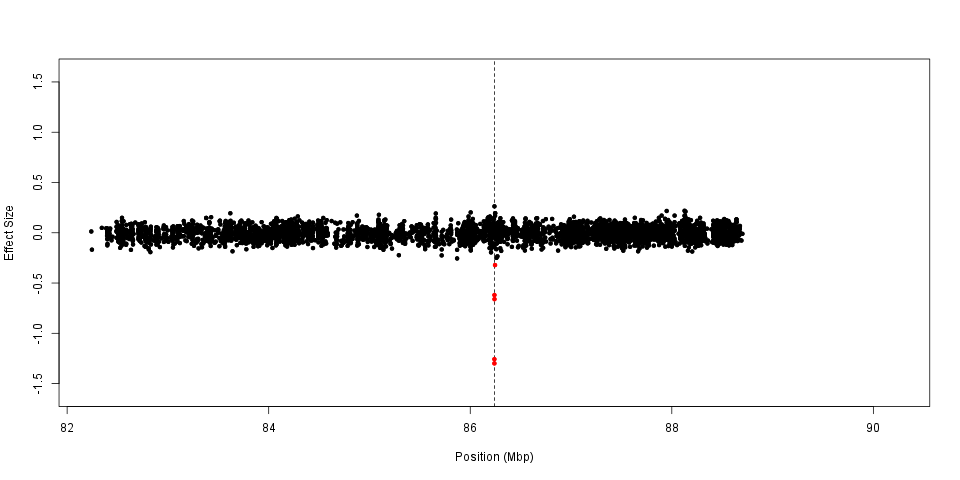

Supplement: Additional file 9: Figure S8 — Effect size for the association between most significant SNPs from the GWAS of the 10 most heritable methylation probes and the surrounding DNA methylation probes in the surrounding 8Mbp window. (a) rs1374010 - chromosome 13, (b) rs73351675 - chromosome 17, (c) rs11763218 - chromosome 7, (d) rs34212454 - chromosome 21, (e) rs748554 - chromosome 16, (f) rs4075355 - chromosome 14, (g) rs2040847 - chromosome 17, (h) rs10021525 - chromosome 4, (i) rs10848167 - chromosome 12. [file gb-2014-15-5-r73-S9.zip › 3001_6051837451123248_MOESM14_ESM.png]

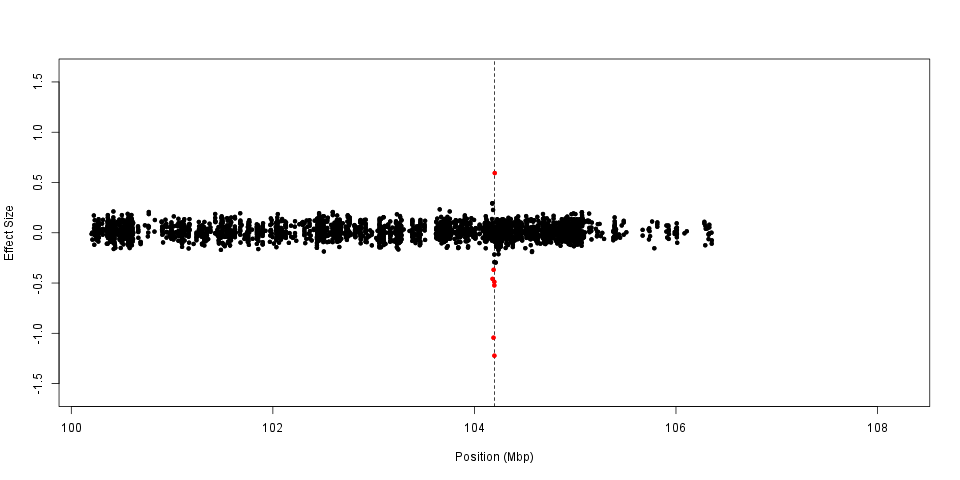

Supplement: Additional file 9: Figure S8 — Effect size for the association between most significant SNPs from the GWAS of the 10 most heritable methylation probes and the surrounding DNA methylation probes in the surrounding 8Mbp window. (a) rs1374010 - chromosome 13, (b) rs73351675 - chromosome 17, (c) rs11763218 - chromosome 7, (d) rs34212454 - chromosome 21, (e) rs748554 - chromosome 16, (f) rs4075355 - chromosome 14, (g) rs2040847 - chromosome 17, (h) rs10021525 - chromosome 4, (i) rs10848167 - chromosome 12. [file gb-2014-15-5-r73-S9.zip › 3001_6051837451123248_MOESM15_ESM.png]

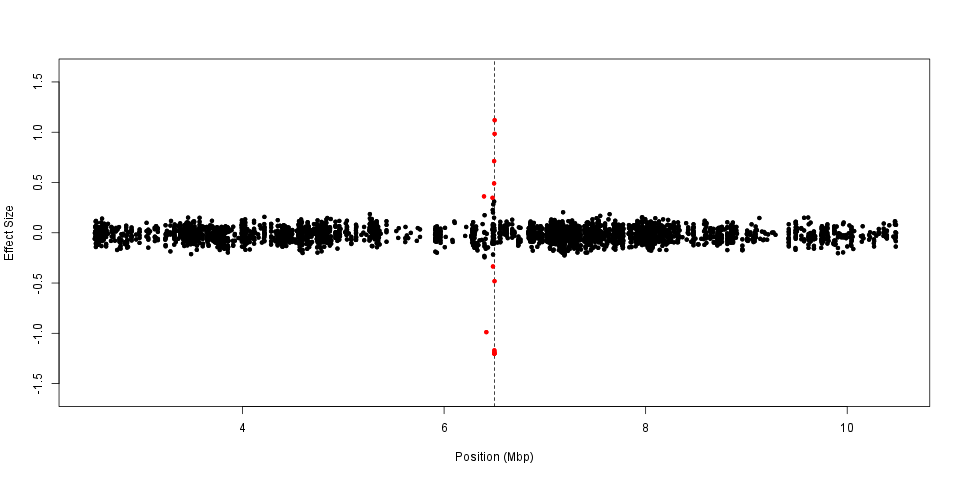

Supplement: Additional file 9: Figure S8 — Effect size for the association between most significant SNPs from the GWAS of the 10 most heritable methylation probes and the surrounding DNA methylation probes in the surrounding 8Mbp window. (a) rs1374010 - chromosome 13, (b) rs73351675 - chromosome 17, (c) rs11763218 - chromosome 7, (d) rs34212454 - chromosome 21, (e) rs748554 - chromosome 16, (f) rs4075355 - chromosome 14, (g) rs2040847 - chromosome 17, (h) rs10021525 - chromosome 4, (i) rs10848167 - chromosome 12. [file gb-2014-15-5-r73-S9.zip › 3001_6051837451123248_MOESM16_ESM.png]

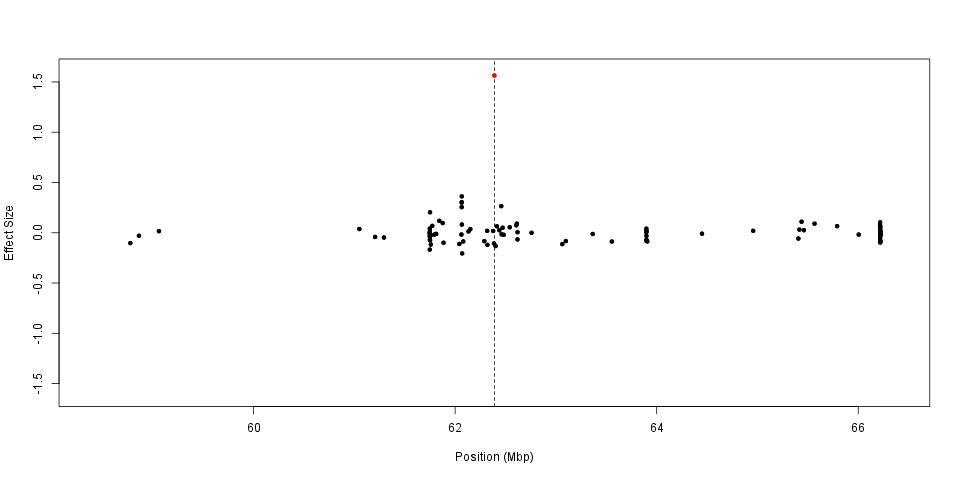

Supplement: Additional file 9: Figure S8 — Effect size for the association between most significant SNPs from the GWAS of the 10 most heritable methylation probes and the surrounding DNA methylation probes in the surrounding 8Mbp window. (a) rs1374010 - chromosome 13, (b) rs73351675 - chromosome 17, (c) rs11763218 - chromosome 7, (d) rs34212454 - chromosome 21, (e) rs748554 - chromosome 16, (f) rs4075355 - chromosome 14, (g) rs2040847 - chromosome 17, (h) rs10021525 - chromosome 4, (i) rs10848167 - chromosome 12. [file gb-2014-15-5-r73-S9.zip › 3001_6051837451123248_MOESM17_ESM.png]

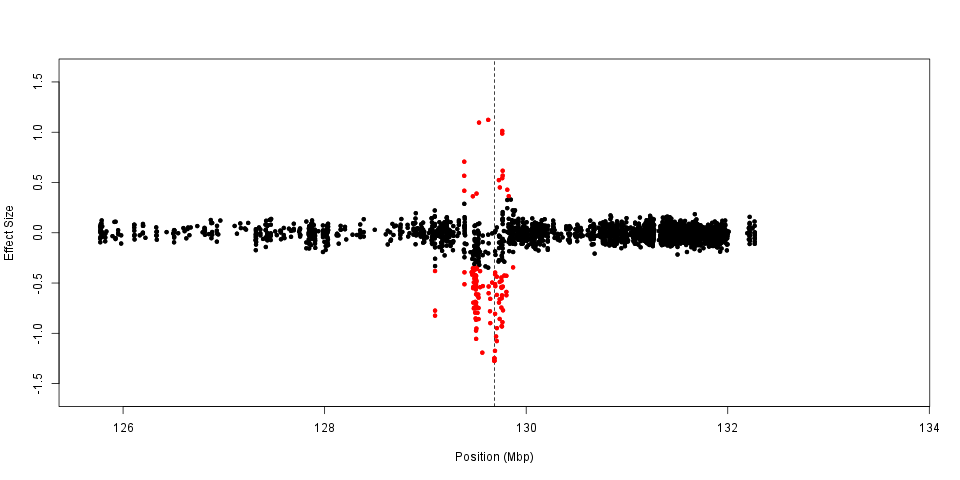

Supplement: Additional file 9: Figure S8 — Effect size for the association between most significant SNPs from the GWAS of the 10 most heritable methylation probes and the surrounding DNA methylation probes in the surrounding 8Mbp window. (a) rs1374010 - chromosome 13, (b) rs73351675 - chromosome 17, (c) rs11763218 - chromosome 7, (d) rs34212454 - chromosome 21, (e) rs748554 - chromosome 16, (f) rs4075355 - chromosome 14, (g) rs2040847 - chromosome 17, (h) rs10021525 - chromosome 4, (i) rs10848167 - chromosome 12. [file gb-2014-15-5-r73-S9.zip › 3001_6051837451123248_MOESM18_ESM.png]

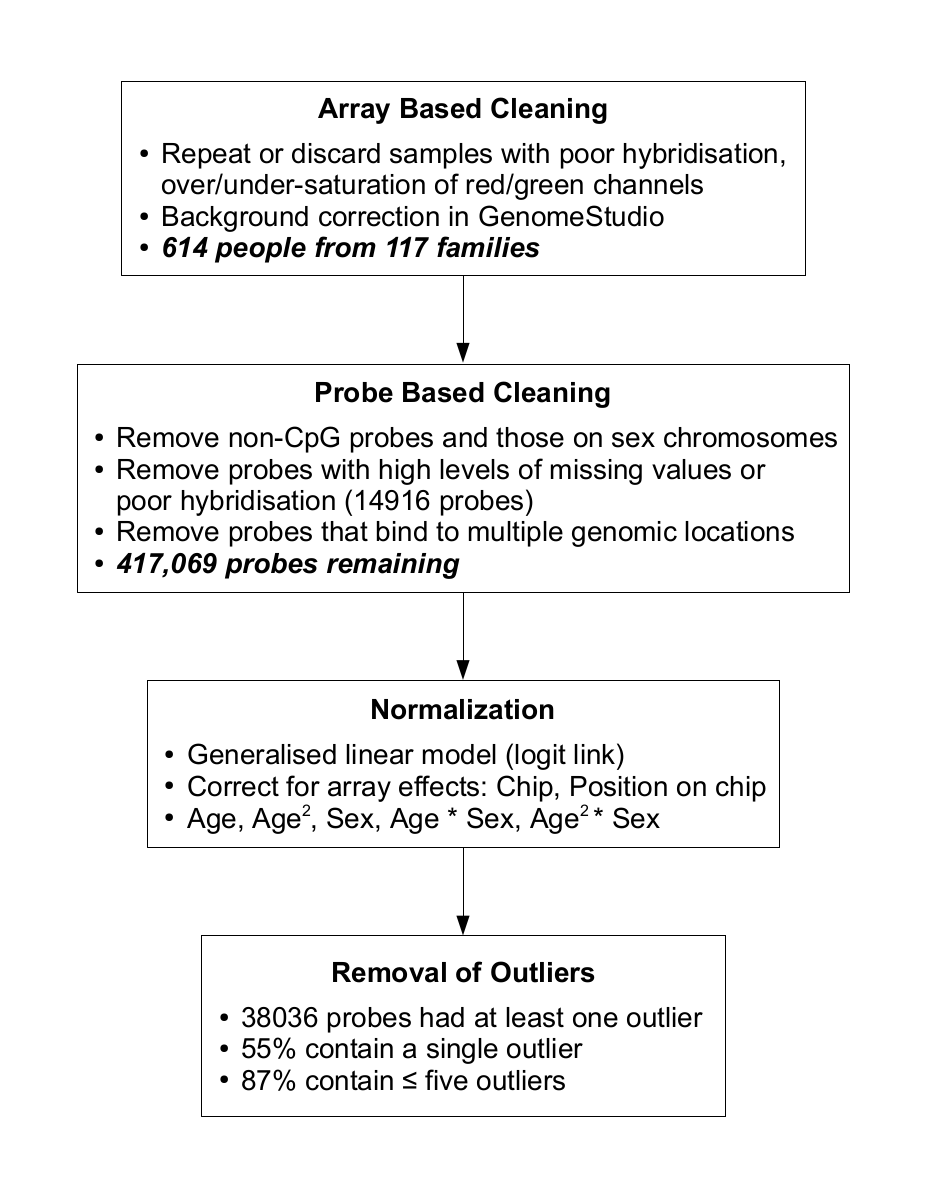

Supplement: Additional file 10: Figure S1 — Flow chart detailing the number of samples and probes removed at each step of the DNA methylation array data cleaning. [file gb-2014-15-5-r73-S10.png]
