# Supplementary material for: Contribution of genetic variation to transgenerational inheritance of DNA methylation
Source: Genome Biol. 2014 May 29;15(5):R73. doi: 10.1186/gb-2014-15-5-r73 (PMC4072933; doi:10.1186/gb-2014-15-5-r73)
Supplement: Additional file 8: Table S2 — Genome-wide significant associations between gene-expression levels and rs111482415. [file gb-2014-15-5-r73-S8.doc]

**Table S2:** Genome-wide significant associations between gene-expression levels and rs111482415.

| Gene | Probe | Location | Effect (S.D.) | P-Value |
| --- | --- | --- | --- | --- |
| HLA-H | ILMN_1666078 | 6:29,859,670 | 0.896 | 2.6 x 10^-51^ |
| HLA-G | ILMN_1656670 | 6:29,906,620 | 0.810 | 5.5 x 10^-41^ |
| HLA-A29.1 | ILMN_2165753 | 6:29,897,513 | 0.661 | 6.4 x 10^-19^ |
| HLA-DRB4 | ILMN_1752592 | 6:32,581,153 | -0.382 | 1.9 x 10^-10^ |
| HCG4 | ILMN_1660923 | 6:29,868,678 | 0.361 | 1.4 x 10^-09^ |
| HLA-A | ILMN_2203950 | 6:29,913,300 | 0.357 | 5.0 x 10^-09^ |
| BTN3A2 | ILMN_1676528 | 6:26,378,113 | 0.349 | 5.4 x 10^-09^ |
| HLA-F | ILMN_1762861 | 6:29,692,132 | -0.336 | 1.9 x 10^-08^ |
| BTN3A2 | ILMN_1700067 | 6:26,368,410 | 0.290 | 6.8 x 10^-07^ |
